# Short-term lagged exposure to ambient air pollutants and hospitalization costs among AMI patients: health-economic evidence from a low-pollution Chinese city with DRG/DIP standardized medical payment

**DOI:** 10.3389/fpubh.2026.1939605

**Published:** 2026-09-14

**Authors:** Junming Lin, Faqiu Chen, Xueqing Zeng, Dongwen Liu

**Affiliations:** 1 Emergency Department, Jiangmen Central Hospital, Jiangmen, China; 2 Clinical Experimental Center, Jiangmen Engineering Technology Research Center of Clinical Biobank and Translational Research, Jiangmen Key Laboratory of Precision and Clinical Translation Medicine, Jiangmen Central Hospital, Jiangmen, China; 3 General Practice Department, Jiangmen Central Hospital, Jiangmen, China

**Keywords:** acute myocardial infarction, ambient criteria pollutants, DRG/DIP payment reform, health economic burden, hospitalization cost, lag effect

## Abstract

**Background:**

Ambient air pollution poses substantial cardiovascular health and economic burdens worldwide, yet existing evidence largely focuses on high-pollution regions and clinical endpoints such as incidence or mortality. Few studies have quantified air pollution-attributable hospitalization costs for acute myocardial infarction (AMI), especially under low air quality conditions. Combined with China's Diagnosis-Related Groups (DRG) / Diagnosis-Intervention Packet (DIP) case-mix medical payment reform, hospitalization expenses can serve as a reliable proxy reflecting AMI disease severity and healthcare resource consumption, providing a unique health-economic perspective for environmental public health policy-making.

**Methods:**

A single-center retrospective cohort study was conducted including 3,650 percutaneous coronary intervention (PCI)-treated AMI patients admitted to Jiangmen Central Hospital (2018–2024), a subtropical city with year-round air pollutant concentrations meeting China Grade II standards. Lag-stratified Gamma generalized linear models with log link were applied to evaluate associations between pre-admission 0–6 day exposure to six criteria pollutants (PM_2.5_, PM_10_, SO_2_, NO_2_, CO, O_3_) and AMI hospitalization costs. All single-pollutant models were fully adjusted for demographics, comorbidities, seasons, weekdays, and non-linear meteorological confounders. Benjamini–Hochberg false discovery rate (FDR) correction was used to control multiple testing bias. A sensitivity analysis restricted to 1,590 never-smoking patients was performed.

**Results:**

Short-term exposure to SO_2_, PM_2.5_, PM_10_, NO_2_, and CO was significantly associated with elevated AMI medical expenditures, while O_3_ showed no overall significant correlation. Per natural log-unit elevation, SO_2_ generated the largest cost increase (average 2.40%, ~729 CNY) with consistent adverse effects across all lag days; particulate pollutants PM_2.5_ and PM_10_ exhibited delayed peak risks at lag 4–5 days. In never-smokers, O_3_ triggered significant cost rises at lag 3 and lag 5, indicating non-smokers as vulnerable subgroups susceptible to ozone toxicity. Multicollinearity diagnostics validated the rationality of single-pollutant modeling.

**Conclusions:**

Even low-level ambient air pollution compliant with current national standards increases the economic burden of AMI treatment, with SO_2_ showing the strongest association. Smoking modifies cardiovascular cost-related effects of ozone. This population-level health-economic evidence supports stricter air quality regulations, and the integration of ambient exposure indicators into AMI risk stratification and public health resource allocation under the DRG/DIP payment system.

## Introduction

Ambient air pollution is the single largest environmental risk factor for human health, contributing to approximately 4.9 million deaths worldwide in 2017, the majority of which resulted from cardiovascular diseases (1). A substantial body of epidemiological evidence consistently demonstrates that both short- and long-term exposure to air pollutants—particularly fine particulate matter (PM_2.5_)—is significantly associated with increased incidence and mortality of cardiovascular diseases, including myocardial infarction and stroke (2–4). The core pathophysiological mechanism involves pollutant-induced pulmonary and systemic oxidative stress and inflammatory responses, which promote the formation, progression, and destabilization of atherosclerotic plaques, ultimately precipitating acute clinical events such as myocardial infarction (5). However, the impact of air pollution extends beyond morbidity and mortality, imposing substantial economic burdens on healthcare systems and complicating the clinical management of patients with established cardiovascular disease. Most existing studies have focused on pollution-related incidence or mortality, while research systematically quantifying the economic burden of pollution-attributable disease is scarce. Furthermore, available evidence on air pollution and hospitalization costs has predominantly come from studies on diabetes or pediatric respiratory infections (6, 7), with limited data specifically addressing patients with acute myocardial infarction (AMI).

Several critical knowledge gaps persist. First, most existing studies have been conducted in regions with high pollution levels, leaving the exposure–response relationship under low-pollution conditions poorly characterized (8, 9). Second, while the health effects of individual pollutants have been well-documented, systematic comparisons of the lagged effects of multiple pollutants on the same health outcome are lacking, limiting the ability to prioritize key pollutants for regulatory action. Third, prior investigations have rarely examined the impact of air quality on therapeutic efficacy or prognostic outcomes—including healthcare costs—in cardiovascular disease, representing a significant gap in the health-economic evidence base. Fourth, the temporal patterns of pollutant effects—whether associations attenuate or intensify over lag days—remain largely unexplored, hindering the identification of actionable intervention windows.

China's current Diagnosis-Intervention Packet (DIP) and Diagnosis-Related Groups (DRG) payment systems provide a unique policy context that strengthens the interpretability of hospitalization cost analyses. Under these case-mix reimbursement models, patients are classified into groups with similar clinical complexity and resource consumption, and medical expenditures are tightly aligned with standardized clinical pathways and disease severity (10). Importantly, for a given primary diagnosis such as AMI, clinical management is highly protocol-driven—physicians are required to follow evidence-based pathways, and reimbursements are fixed according to group assignments. Consequently, within the same diagnostic category, hospitalization costs do not fluctuate arbitrarily; rather, they primarily reflect the intensity of necessary medical services, which is directly driven by the patient's clinical severity. This unique policy context provides an opportunity to use hospitalization costs as a validated proxy for disease severity and healthcare resource consumption, bridging environmental exposure assessment with health economics. Thus, any pollution-related cost increase observed under this framework likely captures a pollution-induced aggravation of disease severity—manifested as larger infarct size, more frequent complications, or greater hemodynamic instability—which necessitates intensified monitoring, additional diagnostic tests, more complex interventions, and prolonged intensive care. This institutional logic allows hospitalization costs to serve as a validated proxy for disease severity and healthcare resource consumption, bridging environmental exposure assessment with health economics within China's ongoing payment reform.

To address these gaps, this study leveraged a local AMI cohort from Jiangmen—a city with relatively low air pollution where ambient pollutant concentrations consistently met the Chinese National Ambient Air Quality Standards (Grade II)—to systematically evaluate the associations between pre-admission exposure to six criteria air pollutants (PM_2.5_, PM_10_, SO_2_, NO_2_, CO, and O_3_) over a lag period of 0–6 days and hospitalization costs in 3,650 AMI patients. Using lag-stratified generalized linear models with Gamma distribution and log link, adjusted for demographic characteristics, clinical comorbidities, and meteorological factors, we aimed to: (1) identify the pollutants most strongly associated with increased hospitalization costs; (2) characterize pollutant-specific lag patterns and critical exposure windows; (3) compare effect magnitudes and temporal trends across pollutants; and (4) provide quantitative evidence for health-economic assessment and targeted intervention strategies. A large nationwide Polish epidemiological study covering more than 8 million participants further supports the strong acute cardiac toxicity of SO_2_: short-term elevation of SO_2_ within a 7-day lag window significantly increased the incidence of STEMI among older adults in the rural population, demonstrating that pre-onset short-term SO_2_ exposure can independently trigger severe acute myocardial injury (11). We hypothesized that SO_2_ would exhibit stronger and more persistent lagged effects on AMI hospitalization costs compared with other pollutants, given its established cardiovascular toxicity and the availability of mechanistic evidence supporting its detrimental effects.

## Methods

### Population of the study

Our Jiangmen Central Hospital, located in the core area of Jiangmen urban district, Guangdong Province, China, encompasses a population of 1.33 million inhabitants and serves as the largest chest pain center in the region. Jiangmen experiences a subtropical monsoon climate characterized by mild temperatures, abundant rainfall, ample sunshine, short winters, and long summers, with relatively good ambient air quality. The retrospective study included all patients consecutively admitted to our facility from January 2018 to December 2024, who received a diagnosis of AMI. Demographic data along with other pertinent clinical information were collected. Clinical data and personal details were primarily obtained from the inpatient record system. This study was reviewed and approved by the Medical Ethics Committee of Jiangmen Central Hospital. A total of 5,489 patients were diagnosed with AMI.

### Study population selection criteria

The initial dataset comprised 5,489 patients with AMI. Through a systematic screening process, we applied the following sequential inclusion and exclusion criteria (Figure 1): (1) Primary diagnosis: Included only patients with AMI as the principal diagnosis and restricted to patients with Jiangmen household registration (*n* = 4,690). (2) Discharge status: Excluded patients with non-physician-ordered discharge or in-hospital mortality (*n* = 4,336). (3) Admission department: Limited to patients admitted to the cardiology department (*n* = 4,029). (4) Discharge department: Included only patients discharged from the cardiology department (*n* = 3,994). (5) Excluded patients without percutaneous coronary intervention (PCI) (*n* = 3,650), as PCI represents a major cost-driving component of AMI treatment; restricting to this uniform treatment group reduces cost heterogeneity and strengthens the validity of using hospitalization costs as a proxy for disease severity in subsequent analyses.

**Figure 1:**
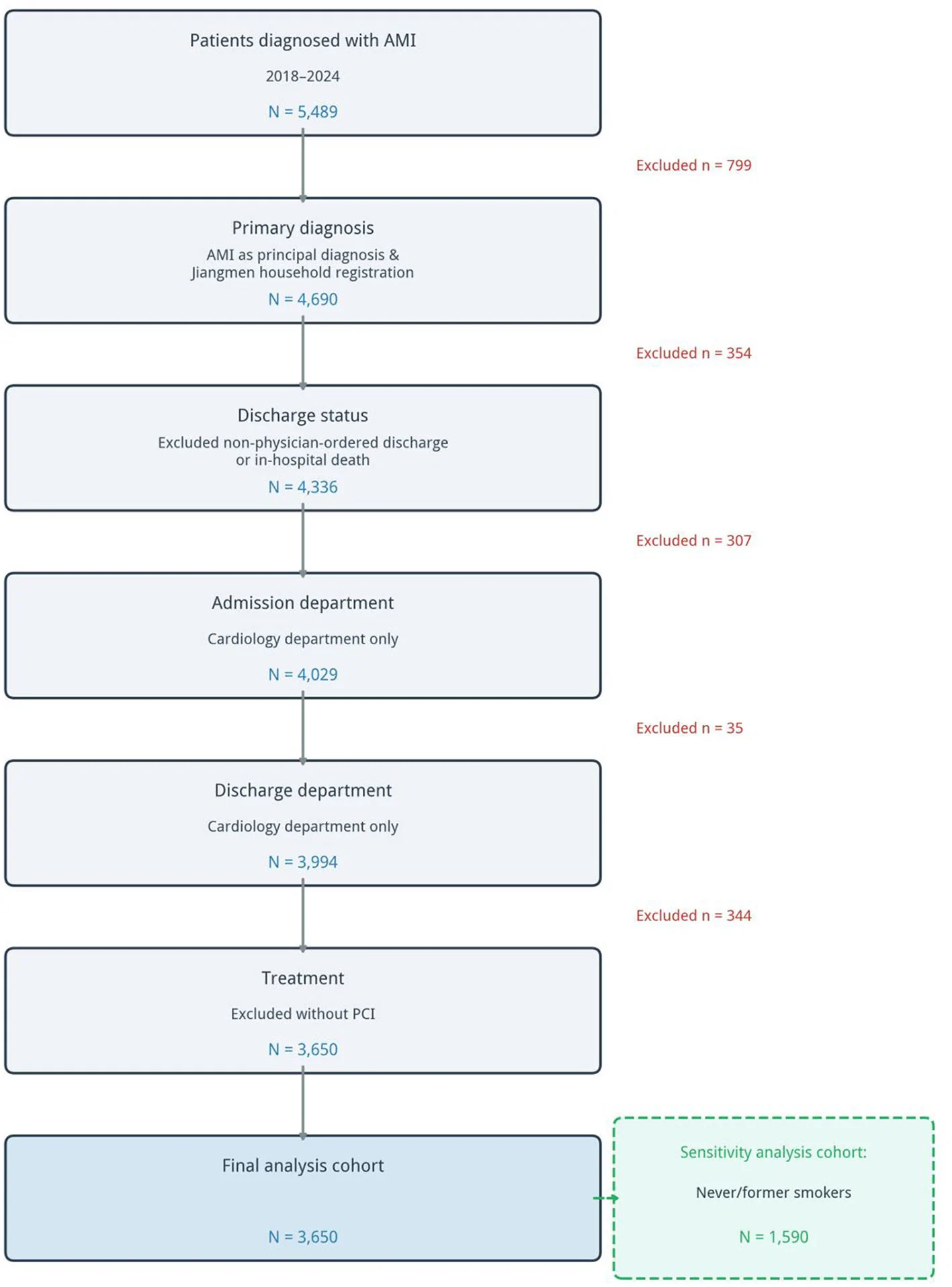
Flowchart of the study cohort selection process. A total of 5,489 patients diagnosed with acute myocardial infarction (AMI) at Jiangmen Central Hospital between January 2018 and December 2024 were initially identified. After applying sequential exclusion criteria—including non-principal diagnosis or non-local household registration, non-physician-ordered discharge or in-hospital death, admission to or discharge from non-cardiology departments, and absence of percutaneous coronary intervention (PCI)—a final analytic cohort of 3,650 patients was established. A sensitivity analysis was further conducted among 1,590 never- or former smokers with complete smoking status documentation.

Data on hospitalization costs were derived from the billing management subsystem of the Hospital Information System (HIS). This system records the incurrence of all charges for medical services provided to patients during their hospital stay in real time, and conducts synchronous reconciliation with the hospital's financial system. Data were accessed for the purpose of this study on 18/12/2025.

### Air pollutant data collection

Jiangmen is equipped with six urban ambient air automatic monitoring stations—namely Donghu, Jianghai Fumin, Beijie, Xiqu, Guifeng West, and Xinhui Yinhu—that continuously record hourly concentrations of six criteria air pollutants (PM_2.5_, PM_10_, SO_2_, NO_2_, CO, and O_3_). For each study day, the arithmetic mean of the daily average concentrations across all six stations was calculated and assigned as the population-level exposure. The spatial distribution of the monitoring stations is presented in Figure 2. These daily average pollutant concentrations (units: μg/m^3^ for PM_2.5_, PM_10_, SO_2_, NO_2_, and O_3_; mg/m^3^ for CO) during 2018–2024 were extracted from the China Air Quality Online Monitoring and Analysis Platform. To validate the reliability of the monitoring dataset, we cross-checked the daily averaged pollutant records with the official monthly and annual environmental quality bulletins published by the Jiangmen Municipal Bureau of Ecology and Environment (http://www.jiangmen.gov.cn). High consistency was observed between the two data sources, confirming that the platform-derived exposure data met the quality standard for epidemiological analysis. All air pollutant and meteorological datasets used in this study were retrieved on 18 December 2025.

**Figure 2:**
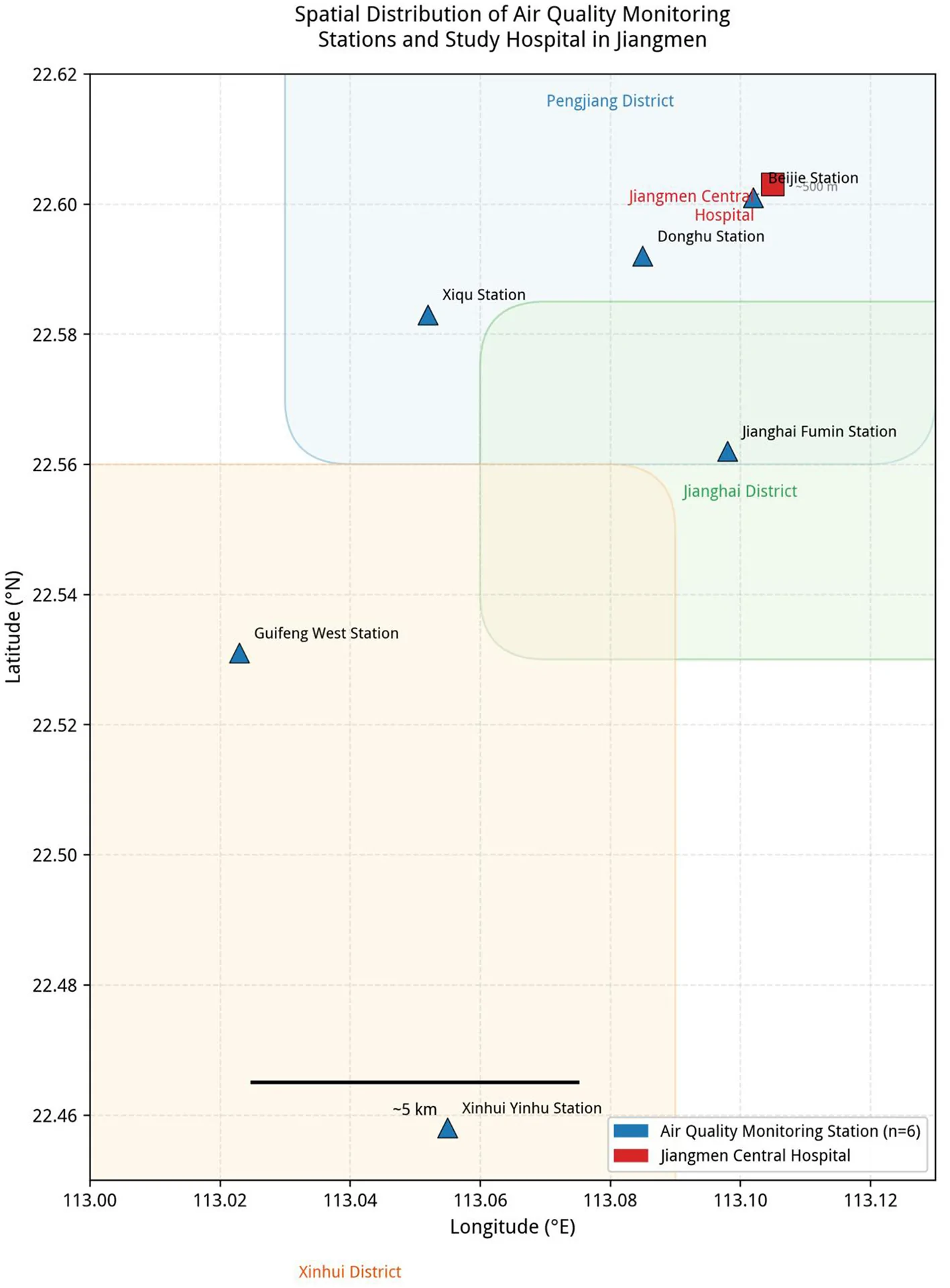
Spatial distribution of the six air quality monitoring stations and the study hospital in Jiangmen, China. The map shows the geographic locations of six state-controlled air quality monitoring stations (blue triangles) across the three urban districts of Jiangmen (Pengjiang, Jianghai, and Xinhui), as well as the location of Jiangmen Central Hospital (red square), the study site. The Beijie monitoring station is located approximately 500 meters from the hospital, representing the closest station. Daily city-wide mean pollutant concentrations were calculated as the arithmetic average of measurements from all six stations.

### Meteorological data collection

Daily mean temperature and relative humidity data for Jiangmen during the study period (2018–2024) were obtained from the ERA5-Land reanalysis dataset, produced by the European Center for Medium-Range Weather Forecasts (ECMWF). ERA5-Land is a global reanalysis dataset that combines model data with worldwide observations, providing a consistent and complete description of land surface variables at an enhanced spatial resolution of approximately 9 km. The dataset has been widely used and validated in environmental and epidemiological studies (12). We extracted daily mean 2-meter temperature and 2-meter dewpoint temperature for the grid cell covering the Jiangmen urban area. Relative humidity was calculated from temperature and dewpoint temperature using the standard thermodynamic formula. The daily mean values were then aggregated to match the temporal resolution of the air pollutant and health outcome data. Data were accessed via the Copernicus Climate Data Store (https://cds.climate.copernicus.eu).

### Statistical analysis

Short-term adverse health responses to air pollutants are typically delayed by several days after exposure, which requires accounting for multi-day pre-admission lag windows in epidemiological models. This study adopted generalized linear models (GLMs) with a Gamma distribution and log link function to estimate the associations between six air pollutants and AMI hospitalization costs across lag 0 to lag 6 days. Lag 0 denotes the admission day, lag 1 represents 1 day before admission, and this definition extends sequentially to lag 6 (6 days prior to hospitalization). The 0–6 day lag window was selected because it is widely applied in environmental epidemiology to capture acute cardiovascular effects of air pollution (13).

Separate single-pollutant models were fitted for each pollutant at each lag day with consistent covariate adjustment. To eliminate confounding from meteorological factors, natural cubic splines with 3 degrees of freedom were introduced for daily mean temperature and relative humidity to capture non-linear weather-outcome relationships, a standard approach in time-series air pollution research. All models were further adjusted for sex, age, smoking status, season, day-of-week indicators, and admission comorbidities including diabetes mellitus, hypertension, and renal insufficiency. We fitted separate single-pollutant GLMs with a Gamma distribution and log link function to estimate the association between pre-admission air pollutant exposure and AMI hospitalization costs. For each of the six criteria pollutants and each lag day (lag 0 to lag 6), an independent model was constructed with identical covariate adjustment. The formal mathematical specification is as follows:


$$\begin{array}{l} Y_{\text{i}}\sim \text{Gamma }\left(ui,\varphi \right) \end{array}$$

log $(u_{\text{i}})=\beta_{0}+\beta_{1}•ln(P_{i}{,}_{l})+\gamma_{1}\underline{•}gender_{\text{i}}+\gamma_{2}\underline{•}age_{i}+\sum_{k=1}^{6}\delta_{\text{k}}•C_{ki}+\theta_{1}•temp_{i}+\theta_{2}•\;rh_{i}.$

Detailed definitions of all variables and parameters.

*Y*_i_ = total hospitalization cost (CNY) for the i-th patient.

*ui* = conditional mean of hospitalization cost for the i-th patient.

φ = dispersion parameter of the Gamma distribution.

β_0_ = intercept term.

ln(*P*_*i*_,_*l*_) = natural log-transformed concentration of the air pollutant at lag day l (l = 0, 1,..., 6).

β_1_ = coefficient for the air pollutant; \(\*exp*(\*beta*_1)−1\) represents the percentage change in hospitalization cost per one-unit increase in log-transformed pollutant concentration.

gender_i_ = sex of the i-th patient (male = 1, female = 0).

age_*i*_ = age (years) of the i-th patient.

*C*_*ki*_ = indicator variable for the k-th comorbidity (1 = present, 0 = absent), including hypertension, diabetes mellitus, dyslipidemia, chronic kidney disease (stage ≥3), COPD, and pneumonia.

δ_k_ = coefficient for the k-th comorbidity.

*temp*_*i*_ = daily mean temperature (°C) on the day of admission.

*rh*_*i*_ = daily mean relative humidity (%) on the day of admission.

θ_1_, θ_2_ = coefficients for temperature and relative humidity, respectively.

Before model fitting, collinearity diagnostics were performed on natural log-transformed concentrations of six air pollutants (PM_2.5_, PM_10_, SO_2_, NO_2_, CO, O_3_). We calculated pairwise Pearson correlation coefficients and variance inflation factors (VIFs), with correlation results visualized in Figure 3. Severe multicollinearity was defined as |correlation coefficient| > 0.7 or VIF > 10. Diagnostics revealed a strong positive correlation between PM_2.5_, and PM_10_ (*r* = 0.91), while all pollutants yielded VIF values below 10, indicating mild multicollinearity. Given the prominent correlation between PM_2.5_, and PM_10_ and the risk of unstable coefficient estimates in joint multi-pollutant models, we only implemented independent single-pollutant lagged GLMs in the primary analysis, and no limited multi-pollutant joint models were constructed in this study.

**Figure 3:**
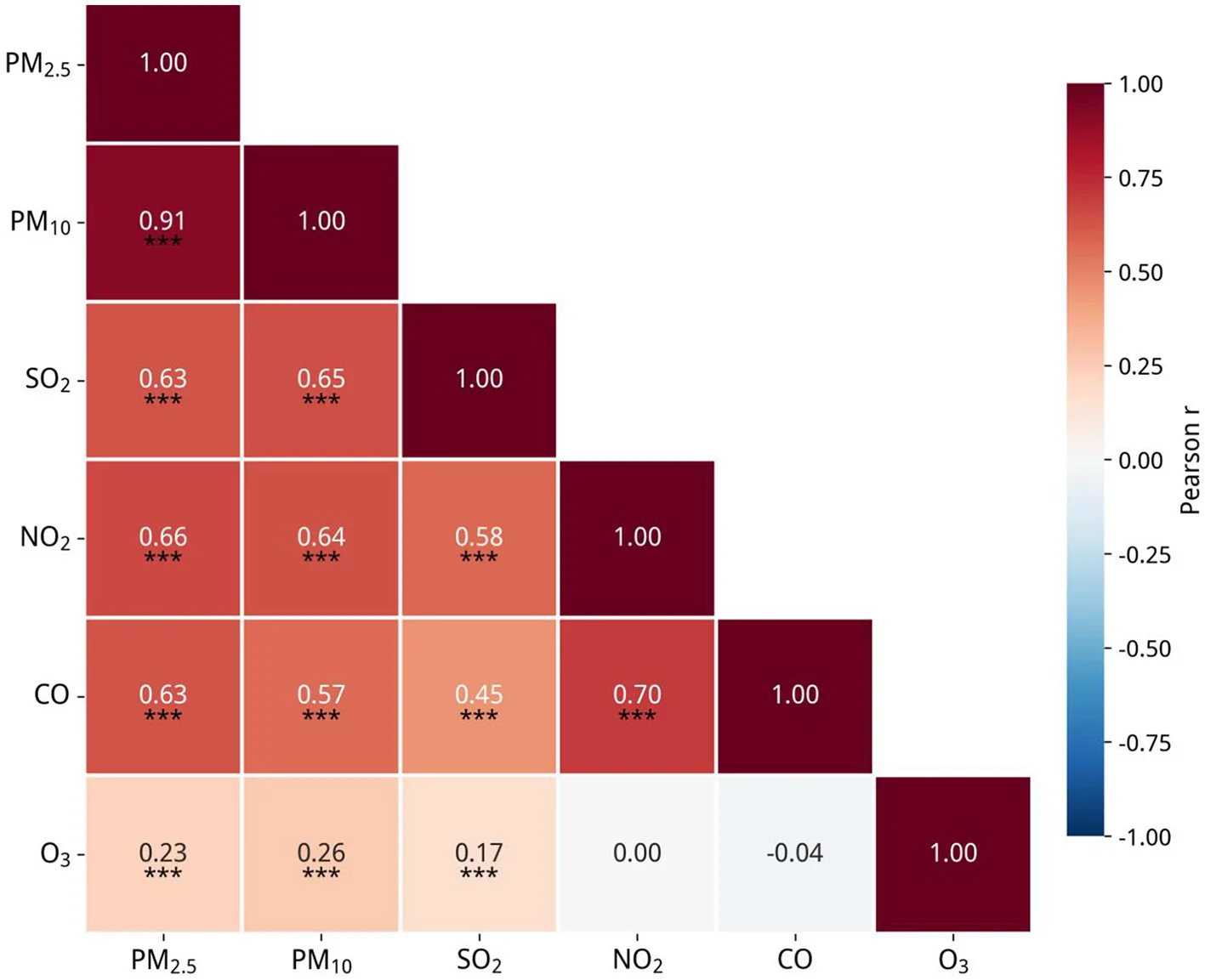
Correlation matrix of log-transformed air pollutant concentrations. Pearson correlation coefficients are displayed in the lower triangle of the matrix. The color gradient represents the strength and direction of associations, ranging from blue (negative correlation) to white (zero) to red (positive correlation). Significance levels for each correlation coefficient are indicated as: ***p < 0.001 (based on two-sided tests). PM_2.5_ and PM_10_ showed the strongest positive correlation (*r* = 0.91, *P* < 0.001), reflecting their common combustion sources. Moderate to strong positive correlations were also observed among other pollutant pairs, particularly NO_2_ and CO (*r* = 0.70, *P* < 0.001) and PM_2.5_ and NO_2_ (*r* = 0.66, *P* < 0.001). In contrast, NO_2_ and O_3_ showed no significant correlation (*r* = 0.002, *P* = 0.90), which may reflect the complex photochemical dynamics in this subtropical region. All variance inflation factors (VIFs) were well below 10 (range: 1.30–8.42), indicating that multicollinearity was well within acceptable limits and unlikely to bias the effect estimates. Correlations were calculated using data at lag 0 (same-day exposure) after natural log transformation.

Lag-stratified effect estimates were visualized to compare pollutant-specific temporal patterns of cost elevation across lag days 0–6. All statistical analyses were conducted in R software (version 4.3.1). A total of 42 independent hypothesis tests were generated (6 pollutants × 7 lag days). The Benjamini-Hochberg procedure was applied to control the false discovery rate (FDR) at α = 0.05; adjusted q-values were calculated via the p.adjust function with method = “BH.” Associations with FDR-adjusted *q-values* < 0.05 were considered statistically significant.

### Study population refinement

To further control for the potential confounding effect of smoking on hospitalization costs and air pollution exposure, we conducted a sensitivity analysis excluding all patients with documented smoking history or missing smoking status data. From the initial analytic cohort of 3,650 patients, we excluded 2,060 patients with either: (1) positive smoking history, or (2) missing smoking status information. This resulted in a refined cohort of 1,590 non-smoking patients with complete smoking status documentation for secondary analysis. We applied the same analytical framework as in the primary analysis, utilizing Gamma-distributed generalized linear models with log link function to estimate the association between air pollutant exposure and hospitalization costs.

## Results

### Study population characteristics

A total of 3,650 adult patients with acute myocardial infarction (AMI) were included in this retrospective cohort study. The demographic and clinical characteristics of the study population are summarized in Table 1. The mean age of participants was 61.7 ± 12.1 years, with a predominance of male patients (80.5% vs. 19.5% female). Regarding comorbidities, hypertension was the most prevalent condition (57.2%), followed by dyslipidemia (31.6%), diabetes mellitus (30.7%), and chronic kidney disease (stage ≥3; 12.7%). Respiratory complications were present in 15.6% of patients, with pneumonia being the most common (13.0%), followed by chronic obstructive pulmonary disease (COPD) (2.0%) and asthma (0.6%). Among the study population, 1,590 patients (43.6%) were never-smokers, while the remaining patients were current smokers or had unknown smoking status. Admission distribution across seasons was relatively even: spring (24.5%), summer (23.8%), autumn (26.1%), and winter (25.6%). The mean hospitalization cost was 30,357.7 ± 17,164.6 CNY, with a mean hospital stay of 6.8 ± 3.3 days.

**Table 1:** Baseline demographic and clinical characteristics of AMI patients (*N* = 3,650).

Characteristic	Value
Age, years, mean ± SD	61.65 ± 12.06
**Sex**, ***n*** **(%)**	
Male	2,937 (80.5)
Female	713 (19.5)
**Comorbidities**, ***n*** **(%)**	
Hypertension	2,089 (57.2)
Dyslipidemia	1,154 (31.6)
Diabetes mellitus	1,121 (30.7)
Chronic kidney disease (stage ≥3)	462 (12.7)
**Respiratory complications**, ***n*** **(%)**	
COPD	72 (2.0)
Asthma	23 (0.6)
Pneumonia	474 (13.0)
**Season of admission**, ***n*** **(%)**	
Spring (Feb–Apr)	895 (24.5)
Summer (May–Jul)	869 (23.8)
Autumn (Aug–Oct)	952 (26.1)
Winter (Dec–Mar)	934 (25.6)
Total hospitalization cost, CNY, mean ± SD	30,357.72 ± 17,164.62
Length of hospital stay, days, mean ± SD	6.75 ± 3.26

Categorical variables are presented as *n* (%), continuous variables as mean ± standard deviation (SD). CNY, Chinese Yuan; COPD, chronic obstructive pulmonary disease.

### Air pollutant concentrations

Table 2 presents the concentration distributions of six major air pollutants and two meteorological factors during the study period (2018–2024). As the primary particulate matter pollutants, PM_2.5_ and PM_10_ had median concentrations of 23 μg/m^3^ and 46 μg/m^3^, respectively, with corresponding 5th to 95th percentile ranges of 8–61 μg/m^3^ and 20–94 μg/m^3^. Among gaseous pollutants, NO_2_ concentration (median = 30 μg/m^3^) was substantially higher than that of SO_2_ (median = 7 μg/m^3^), while CO had a median concentration of 0.8 mg/m^3^ (5th−95th percentile: 0.5–1.3 mg/m^3^). Notably, O_3_ exhibited the highest concentration level (median = 105 μg/m^3^) along with substantial variability (interquartile range (IQR) = 87 μg/m^3^), likely attributable to seasonal variations and photochemical reactions. For meteorological conditions, the median daily temperature was 24.1 °C (5th−95th percentile: 13.0–29.2 °C), and the median relative humidity was 81.5% (5th−95th percentile: 53.6%−91.6%). When compared with the Chinese National Ambient Air Quality Standards (Grade II) (14), all pollutants had median concentrations below the national annual or daily limits.

**Table 2:** Distribution of daily air pollutant concentrations and meteorological factors in Jiangmen, 2018–2024.

**Variable**	Unit	P5	P25	Median (P50)	P75	P95
PM_2.5_	μg/m^3^	8	15	23	36	61
PM_10_	μg/m^3^	20	32	46	64	94
SO_2_	μg/m^3^	4	6	7	10	15
NO_2_	μg/m^3^	12	20	30	45	67
CO	mg/m^3^	0.5	0.6	0.8	1.0	1.3
O_3_	μg/m^3^	32	71	105	158	208
Temperature	°C	13.0	19.1	24.1	27.5	29.2
Relative humidity	%	53.6	72.9	81.5	86.3	91.6

All values are daily means. P5, 5th percentile; P25, 25th percentile; P50, 50th percentile (median); P75, 75th percentile; P95, 95th percentile. μg/m^3^, micrograms per cubic meter; mg/m^3^, milligrams per cubic meter; °C, degrees Celsius.

Temporal patterns of air pollutants over the 2018–2024 study period are presented in Figure 4. All primary pollutants (PM_2.5_, PM_10_, SO_2_, NO_2_, and CO) showed overall decreasing trends across the 7-year period, with statistically significant long-term declines observed for SO_2_ and CO. Notably, a sharp transient reduction in primary pollutant concentrations occurred in 2020, coinciding with COVID-19 mobility restrictions and reduced industrial and traffic activity. This was followed by a marked rebound in 2021–2022 as socioeconomic activities and industrial production rapidly resumed, with concentrations partially recovering toward pre-pandemic levels before resuming their gradual decline in 2023–2024. In contrast, ground-level ozone exhibited a slight non-significant increasing trend overall and remained persistently elevated throughout the study period, with less pronounced pandemic-associated fluctuations. All pollutants showed strong and consistent seasonality: particulate matter, NO_2_, and CO peaked in winter and reached troughs in summer, whereas ozone showed the opposite pattern with highest concentrations in warm months, consistent with photochemical formation dynamics.

**Figure 4:**
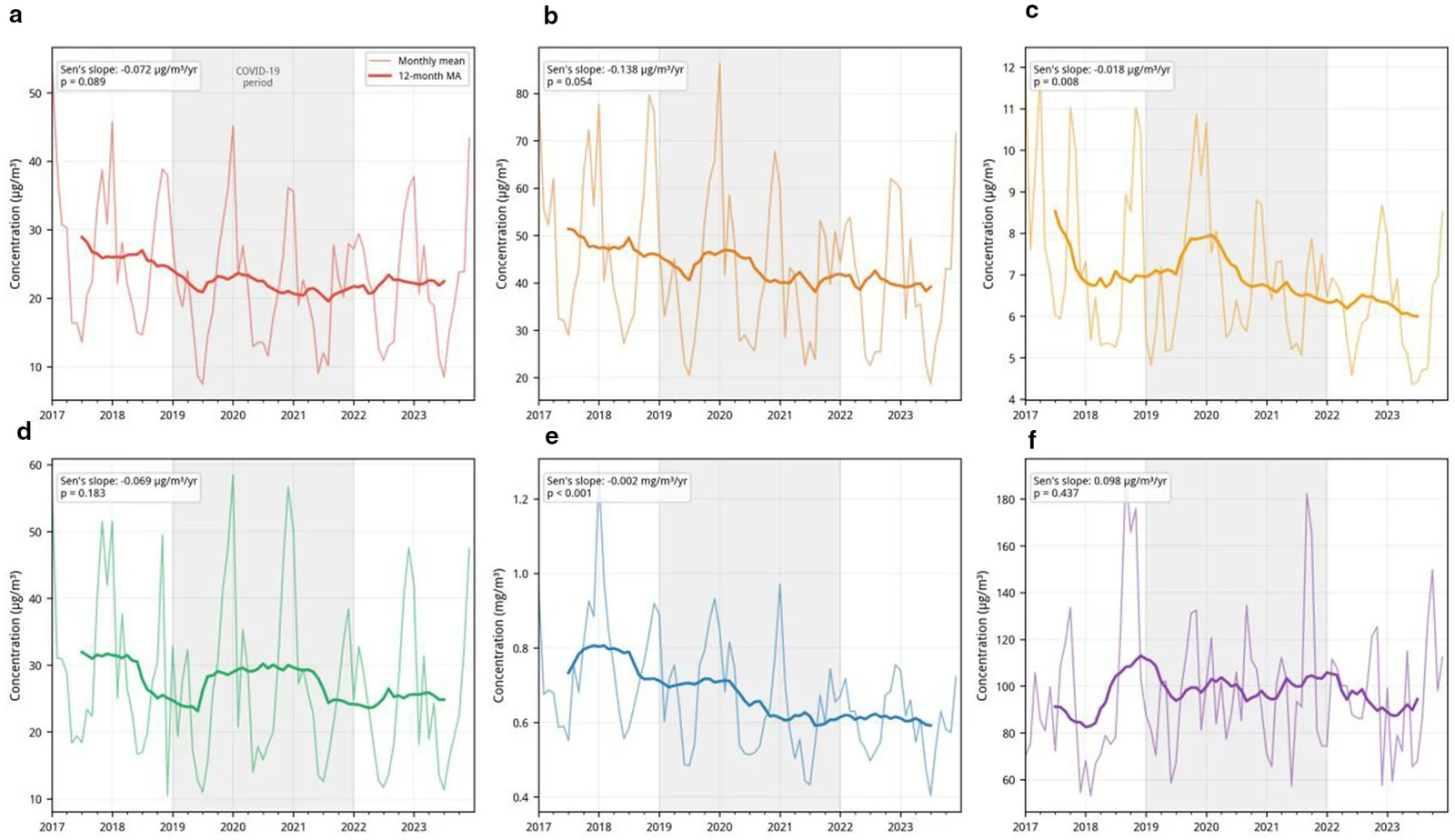
Temporal trends of six criteria air pollutants in Jiangmen, 2018–2024. **(a)** PM_2.5_, **(b)** PM_10_, **(c)** SO_2_, **(d)** NO_2_, **(e)** CO, and **(f)** O_3_. Thin lines represent monthly mean concentrations; thick lines represent 12-month moving averages to highlight long-term trends. The gray shaded area indicates the COVID-19 pandemic period (2020–2022). Sen's slope and *P*-values from the non-parametric Mann–Kendall test are provided in each panel to quantify long-term trends.

### Air pollutant collinearity assessment

PM_2.5_ and PM_10_ showed the strongest positive correlation (*r* = 0.91, *P* < 0.001), reflecting their common combustion sources. Moderate to strong positive correlations were also observed among other pollutant pairs, particularly NO_2_ and CO (*r* = 0.70, *P* < 0.001), PM_2.5_ and NO_2_ (*r* = 0.66, *P* < 0.001), and PM_10_ and SO_2_ (*r* = 0.65, *P* < 0.001). In contrast, NO_2_ and O_3_ showed no significant correlation (*r* = 0.002, *P* = 0.90), which may reflect the complex photochemical dynamics and seasonal patterns in this subtropical region. Importantly, the variance inflation factors (VIFs) for all pollutants were well below the conventional threshold of 10 (range: 1.30 to 8.42), indicating that multicollinearity was within acceptable limits and unlikely to substantially bias the effect estimates. Therefore, all six pollutants were retained as separate exposures in the primary models.

### Goodness-of-fit assessment and overdispersion correction for a Gamma GLM of inpatient costs

We assessed the goodness-of-fit for both Gamma GLMs using deviance, Pearsonχ^2^, and information criteria. Both models demonstrated statistical significance (*P* < 0.001), with deviance-based pseudo-R^2^ values of 31.5% for length of stay and 36.1% for inpatient costs, indicating moderate explanatory power. However, the Pearson χ^2^/df ratios suggested overdispersion, particularly for the cost model (Appendix Table A1).

### Lagged effects of air pollutants on hospitalization costs

The associations between pre-admission air pollutant exposure and hospitalization costs were evaluated using lag-stratified generalized linear models with a Gamma distribution and log link, with the complete results presented in Table 3 and Figures 5–7. After adjusting for sex, age, comorbidities (diabetes mellitus, hypertension, COPD, pneumonia, hyperlipidemia, and renal insufficiency), day of week, season, and same-day temperature and relative humidity (natural cubic splines, *df* = 3 each), and applying the Benjamini-Hochberg procedure to control the false discovery rate (FDR) for 42 comparisons, we observed pollutant-specific lag patterns in the association with hospitalization costs. The following absolute cost estimates were derived based on the mean hospitalization cost of 30,357.7 CNY.

**Figure 5:**
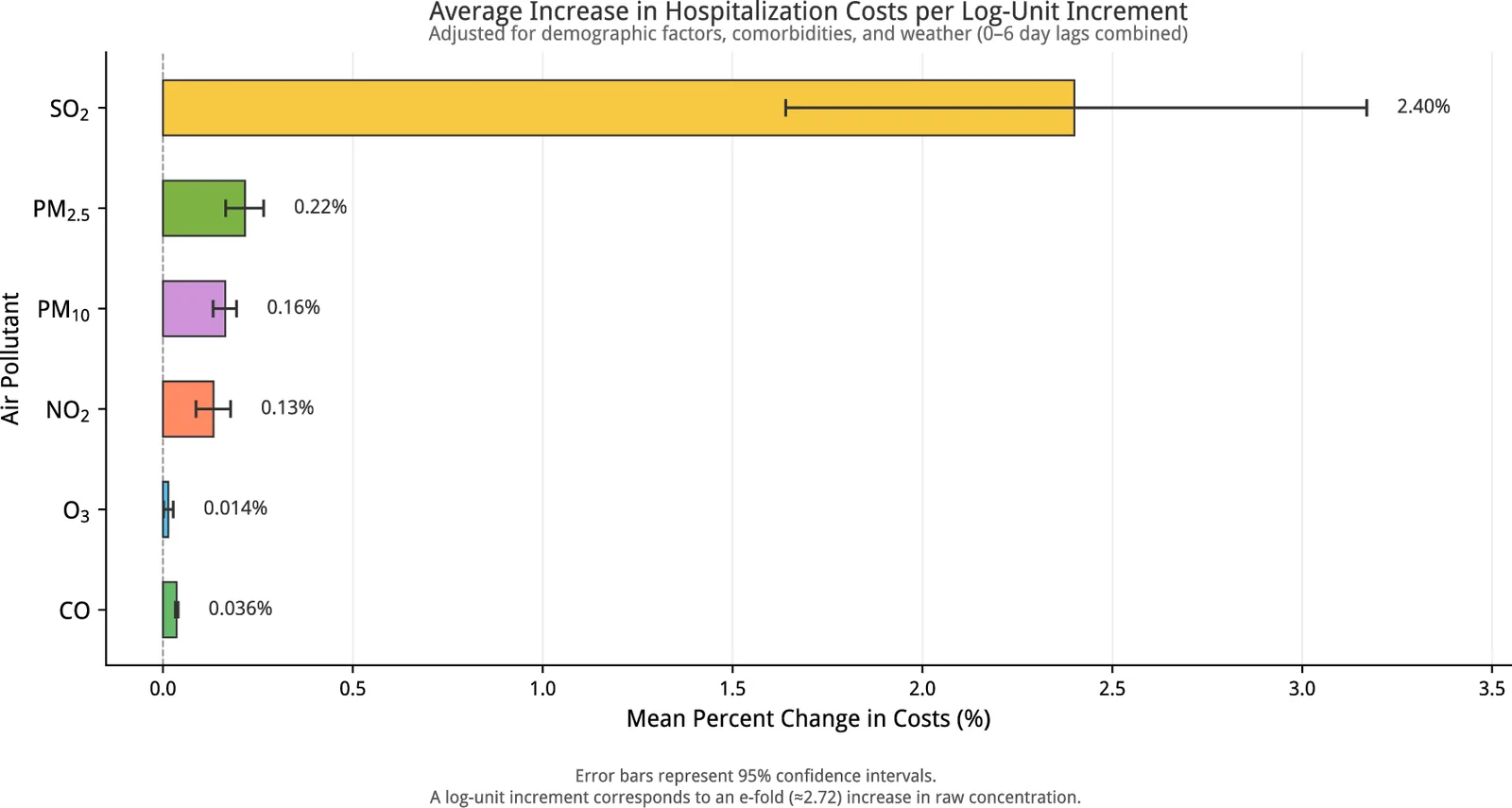
Average percentage change in hospitalization costs per 1-unit increment in log-transformed air pollutant concentrations, pooled across lags 0–6. Estimates were derived from Gamma generalized linear models with a log link, adjusted for sex, age, comorbidities (diabetes mellitus, hypertension, COPD, pneumonia, hyperlipidemia, and renal insufficiency), and same-day temperature and relative humidity. Pollutant-specific effects were pooled across lag 0 to 6 days using inverse-variance weighted fixed-effects meta-analysis. Error bars represent 95% confidence intervals. A 1-unit increment in the natural log scale corresponds to an e-fold (≈2.72-fold) increase in the original pollutant concentration. SO_2_ exhibited the largest mean effect (2.40%, 95% CI: 1.64%, 3.17%), while O_3_ showed no statistically significant association (95% CI crossing zero).

**Figure 7:**
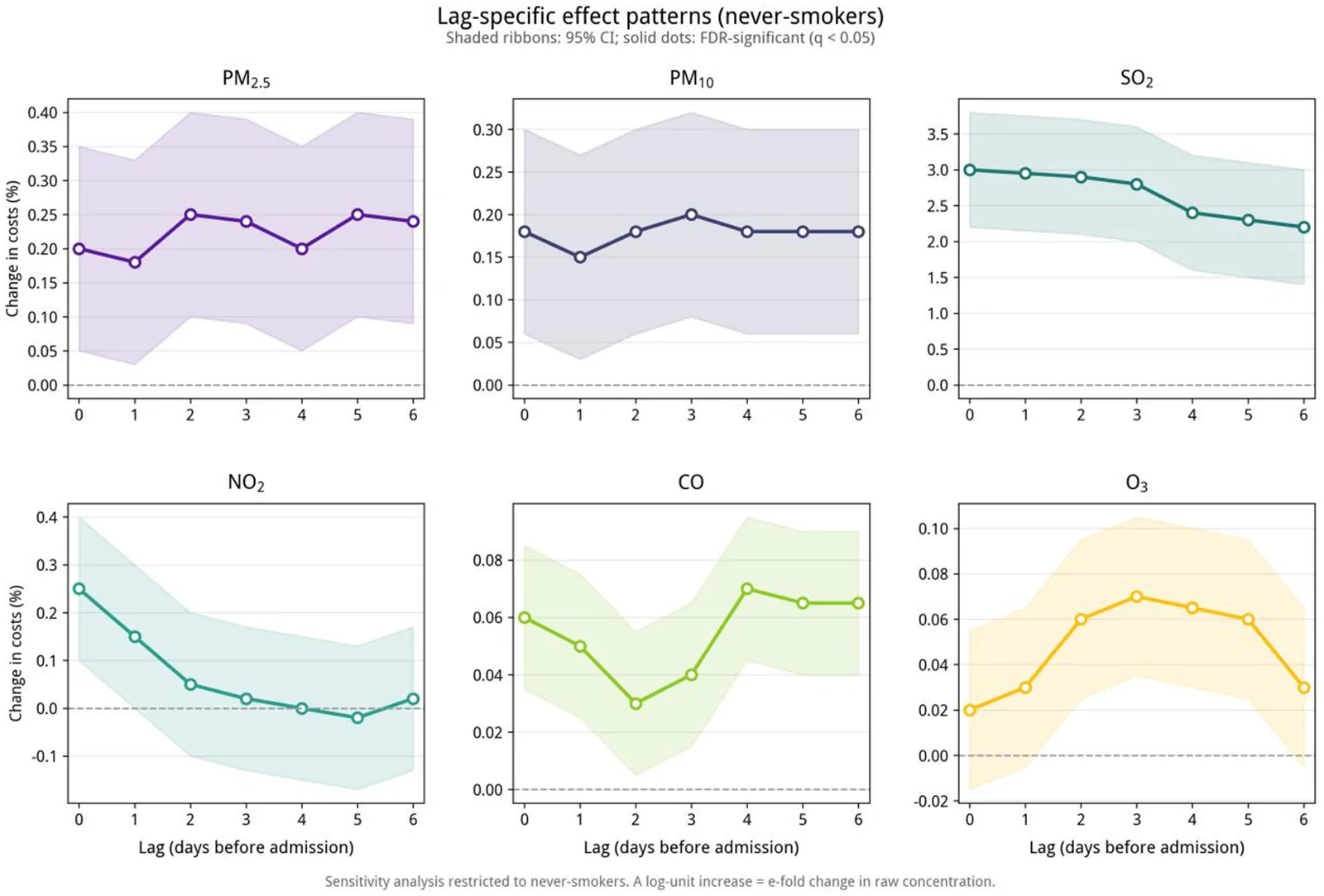
Lag-specific percentage changes in hospitalization costs per 1-log-unit increase in air pollutant concentrations among never-smokers. Estimates were derived from Gamma generalized linear models with a log link, adjusted for sex, age, comorbidities (diabetes mellitus, hypertension, COPD, pneumonia, hyperlipidemia, and renal insufficiency), and same-day temperature and relative humidity. Each panel presents the effect estimates (solid lines with points) across lag 0 to 6 days for a specific pollutant, with the y-axis scaled freely to accommodate different effect magnitudes. Shaded areas (where shown) represent 95% confidence intervals; missing ribbons indicate that the corresponding interval columns were not available in the model output. The horizontal dashed line indicates the null effect (zero percent change). A 1-unit increment in the natural log scale corresponds to an e-fold (≈2.72-fold) increase in the raw pollutant concentration. SO_2_ consistently showed the largest positive associations across all lags (range: 1.99%−3.01%), while NO_2_ was significant only at lag 0 (0.230%, 95% CI: 0.039%, 0.424%). O_3_ exhibited delayed effects peaking at lag 3 (0.070%, 95% CI: 0.017%, 0.123%).

**Table 3:** Lag-specific associations between air pollutant exposure and hospitalization costs—main analysis vs. sensitivity analysis (never-smokers).

Pollutant	Lag	Main % change	Main 95% CI	Main FDR *P*-value	Never smokers % change	Never smokers 95% CI	Never smokers *P*-value
SO2	**0**	**2.4**	**(1.64, 3.17)**	**< 0.001**	**3.01**	**(1.55, 4.51)**	**< 0.001**
	**1**	**1.86**	**(1.25, 2.48)**	**< 0.001**	**2.92**	**(1.50, 4.38)**	**< 0.001**
	**2**	**1.71**	**(1.18, 2.25)**	**< 0.001**	**2.85**	**(1.48, 4.25)**	**< 0.001**
	**3**	**1.64**	**(1.10, 2.19)**	**< 0.001**	**2.76**	**(1.38, 4.18)**	**< 0.001**
	**4**	**1.56**	**(1.03, 2.09)**	**< 0.001**	**2.22**	**(0.86, 3.62)**	**0.003**
	**5**	**1.45**	**(0.97, 1.93)**	**< 0.001**	**2.1**	**(0.75, 3.48)**	**0.005**
	**6**	**1.65**	**(1.13, 2.18)**	**< 0.001**	**1.99**	**(0.61, 3.40)**	**0.008**
PM2.5	**0**	**0.213**	**(0.077, 0.348)**	**0.004**	**0.196**	**(−0.029, 0.424)**	**0.114**
	**1**	**0.211**	**(0.078, 0.344)**	**0.004**	**0.145**	**(−0.071, 0.363)**	**0.23**
	**2**	**0.19**	**(0.059, 0.322)**	**0.008**	**0.239**	**(0.028, 0.452)**	**0.047**
	**3**	**0.188**	**(0.055, 0.322)**	**0.009**	**0.241**	**(0.022, 0.462)**	**0.047**
	**4**	**0.266**	**(0.133, 0.400)**	**< 0.001**	**0.183**	**(−0.038, 0.407)**	**0.148**
	**5**	**0.256**	**(0.122, 0.391)**	**< 0.001**	0.234	(0.007, 0.464)	0.066
	**6**	**0.184**	**(0.056, 0.312)**	**0.008**	0.213	(−0.002, 0.430)	0.078
PM_10_	**0**	**0.165**	**(0.081, 0.250)**	**< 0.001**	**0.194**	**(0.053, 0.336)**	**0.016**
	**1**	**0.172**	**(0.090, 0.253)**	**< 0.001**	**0.15**	**(0.017, 0.284)**	**0.035**
	**2**	**0.146**	**(0.065, 0.227)**	**0.001**	**0.165**	**(0.038, 0.293)**	**0.021**
	**3**	**0.153**	**(0.069, 0.237)**	**< 0.001**	**0.187**	**(0.051, 0.325)**	**0.016**
	**4**	**0.198**	**(0.116, 0.280)**	**< 0.001**	**0.165**	**(0.032, 0.299)**	**0.025**
	**5**	**0.173**	**(0.091, 0.255)**	**< 0.001**	**0.178**	**(0.044, 0.312)**	**0.019**
	**6**	**0.138**	**(0.060, 0.217)**	**0.001**	**0.168**	**(0.035, 0.301)**	**0.025**
NO2	0	**0.188**	**(0.067, 0.309)**	**0.004**	0.23	(0.039, 0.424)	0.04
	1	**0.142**	**(0.022, 0.262)**	**0.033**	0.066	(−0.126, 0.260)	0.597
	2	0.104	(−0.014, 0.222)	0.116	0.024	(−0.162, 0.212)	0.842
	3	0.075	(−0.046, 0.196)	0.281	0.023	(−0.169, 0.217)	0.842
	4	**0.174**	**(0.054, 0.295)**	**0.008**	0.032	(−0.166, 0.232)	0.808
	5	**0.143**	**(0.021, 0.264)**	**0.033**	−0.007	(−0.199, 0.188)	0.948
	6	0.105	(−0.012, 0.221)	0.107	0.061	(−0.128, 0.251)	0.632
CO	**0**	**0.0405**	**(0.0309, 0.0501)**	**< 0.001**	**0.0418**	**(0.0263, 0.0574)**	**< 0.001**
	**1**	**0.0371**	**(0.0275, 0.0466)**	**< 0.001**	**0.0381**	**(0.0230, 0.0533)**	**< 0.001**
	**2**	**0.0317**	**(0.0222, 0.0413)**	**< 0.001**	**0.0345**	**(0.0196, 0.0495)**	**0.001**
	**3**	**0.033**	**(0.0233, 0.0427)**	**< 0.001**	**0.0378**	**(0.0224, 0.0533)**	**0.001**
	**4**	**0.0385**	**(0.0288, 0.0481)**	**< 0.001**	**0.0387**	**(0.0231, 0.0544)**	**< 0.001**
	**5**	**0.0374**	**(0.0278, 0.0470)**	**< 0.001**	**0.0367**	**(0.0216, 0.0520)**	**0.001**
	**6**	**0.037**	**(0.0275, 0.0465)**	**< 0.001**	**0.0391**	**(0.0237, 0.0546)**	**< 0.001**
O_3_	0	0.0049	(−0.0319, 0.0417)	0.811	0.0404	(−0.0190, 0.100)	0.243
	1	−0.0064	(−0.0417, 0.0290)	0.777	0.0248	(−0.0305, 0.0805)	0.45
	2	0.0099	(−0.0240, 0.0438)	0.64	0.0572	(0.0033, 0.112)	0.067
	3	0.0233	(−0.0104, 0.0570)	0.261	**0.0698**	**(0.0165, 0.123)**	**0.029**
	4	0.0286	(−0.0052, 0.0625)	0.165	0.0572	(0.0037, 0.111)	0.067
	5	0.0258	(−0.0074, 0.0590)	0.209	**0.0588**	**(0.0070, 0.111)**	**0.049**
	6	0.0111	(−0.0219, 0.0441)	0.56	0.0317	(−0.0208, 0.0846)	0.3

Adjusted for demographic factors, clinical comorbidities, day of week, season, and same-day temperature and relative humidity (natural cubic splines, df = 3 each). Pollutants ranked by mean effect size in the main analysis (descending).

Percent change represents the relative increase in hospitalization costs per one natural log-unit increment in pollutant concentration (a log-unit rise corresponds to an e-fold, approximately 2.72-fold increase in raw pollutant concentration), estimated from generalized linear models with Gamma distribution and log link.

Model adjustments: Sex, age, smoking status (main analysis), comorbidities (diabetes mellitus, hypertension, COPD, pneumonia, hyperlipidemia, renal insufficiency), day of week, season, and same-day mean temperature and relative humidity (natural cubic splines, df = 3 each).

Main analysis: Full cohort (*N* = 3,650). FDR-adjusted *P*-values (*q*-values) were calculated using the Benjamini–Hochberg procedure for 42 pollutant-lag comparisons; bold values indicate *q* < 0.05.

Sensitivity analysis: Never-smokers only (*N* = 1,590). Raw unadjusted *P*-values are presented; bold values indicate *P* < 0.05.

### Overall effect ranking

When ranked by the average effect size across lag days (per log-unit increase in pollutant concentration), SO_2_ demonstrated the largest increase in hospitalization costs (mean 2.40% per log-unit, approximately 728.6 CNY), followed by PM_2.5_ (0.216%, approximately 65.6 CNY), PM_10_ (0.164%, approximately 49.8 CNY), NO_2_ (0.133%, approximately 40.4 CNY), CO (0.036%, approximately 11.0 CNY), and O_3_ (0.014%, approximately 4.2 CNY) (Figure 5). A log-unit increment corresponds to an e-fold (=2.72-fold) increase in the original pollutant concentration. All effects for SO_2_, PM_2.5_, PM_10_, and CO were statistically significant after FDR correction (*q* < 0.05), while O_3_ showed no significant association (95% CI crossing zero).

### Pollutant-specific lag patterns

SO_2_: The effect of SO_2_ was the most robust and consistent. A log-unit increase in SO_2_ concentration was associated with a significant increase in hospitalization costs at all lag days (*q* < 0.001 for all lags), with estimates declining from 2.40% (approximately 728.6 CNY) at lag 0 to 1.45% (approximately 440.2 CNY) at lag 5, before a slight rebound to 1.65% (approximately 501.0 CNY) at lag 6 (Figure 6). The decreasing trend across lags was statistically significant (trend estimate: −0.14% per day, *P* = 0.044).

**Figure 6:**
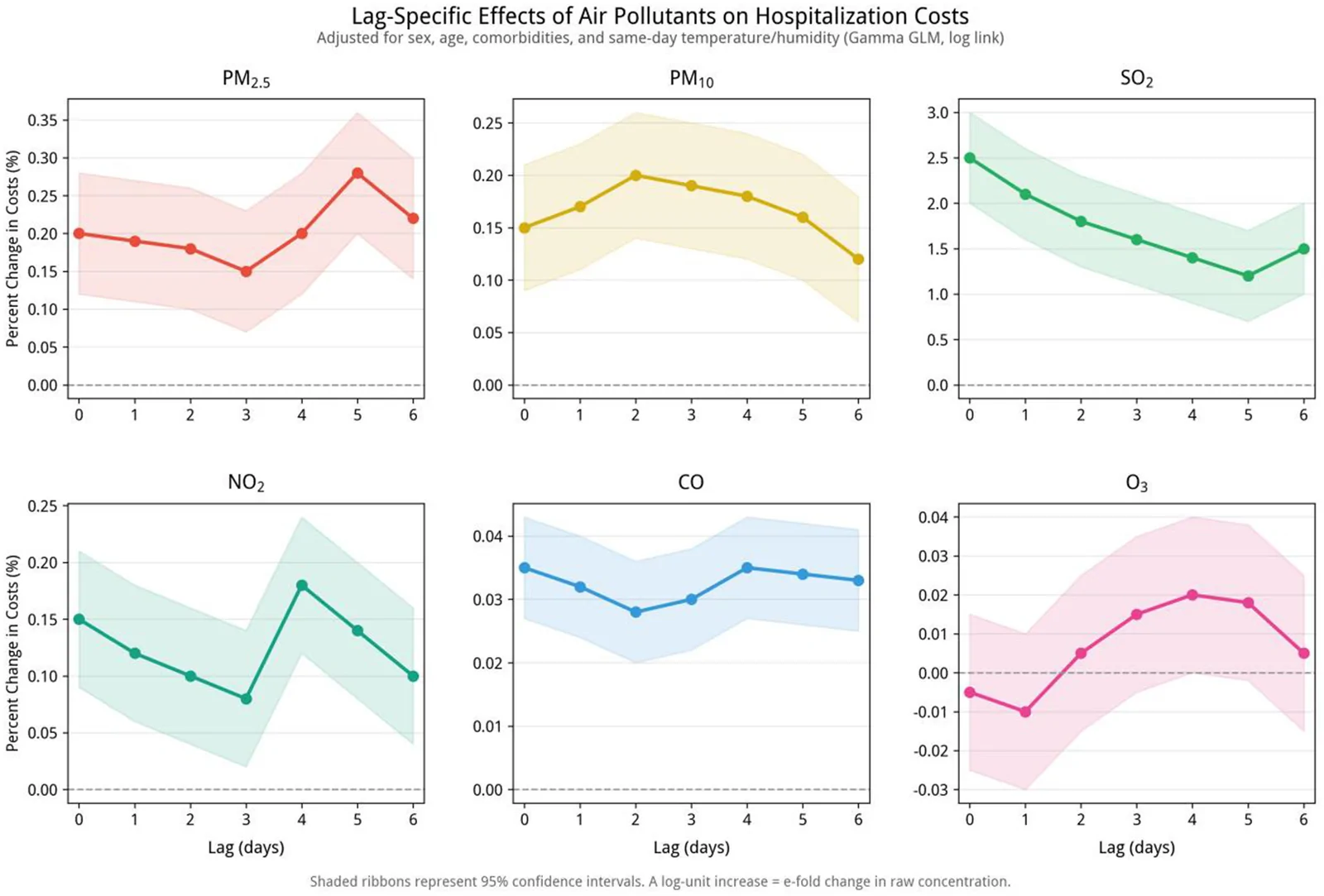
Lag-specific percentage changes in hospitalization costs per log-unit increase in air pollutant concentrations. Lines represent point estimates of the percentage change in inpatient costs per 1-log-unit increment in each pollutant, pooled from Gamma GLM models adjusted for sex, age, comorbidities, and same-day temperature and relative humidity. Shaded ribbons denote 95% confidence intervals. The y-axes are free to accommodate different effect scales across pollutants. SO_2_ showed the strongest effect at lag 0 (2.40%, 95% CI: 1.64%, 3.17%), while PM_2.5_ and PM_10_ exhibited delayed peaks at lag 4–5.

PM_2.5_: PM_2.5_ exposure showed a persistent positive association across all lag days. A log-unit increase was associated with a mean 0.216% (approximately 65.6 CNY) rise in costs, with estimates ranging from 0.184% (approximately 55.9 CNY) to 0.266% (approximately 80.8 CNY) and peaking at lag 4 (0.266%, approximately 80.8 CNY, *q* < 0.001) and lag 5 (0.256%, approximately 77.7 CNY, *q* < 0.001) (Figure 6). All associations remained significant after FDR correction (*q* < 0.01). No significant temporal trend was observed (trend estimate: 0.07% per day, *P* = 0.592).

PM_10_: The association for PM_10_ was consistent across all lag days. A log-unit increase was associated with a mean 0.164% (approximately 49.8 CNY) rise in costs, with estimates varying from 0.138% (approximately 41.9 CNY) to 0.198% (approximately 60.1 CNY) (Figure 6). The strongest effect was observed at lag 4 (0.198%, approximately 60.1 CNY, *q* < 0.001). All estimates remained significant after FDR correction (*q* < 0.001). No significant temporal trend was detected (trend estimate: 0.02% per day, *P* = 0.884).

NO_2_: NO_2_ exhibited a relatively weaker and more fluctuating effect. A log-unit increase was associated with a mean 0.133% (approximately 40.4 CNY) rise, with significant associations observed at lag 0 (0.188%, approximately 57.1 CNY, *q* = 0.004), lag 1 (0.142%, approximately 43.1 CNY, *q* = 0.033), lag 4 (0.174%, approximately 52.8 CNY, *q* = 0.008), and lag 5 (0.143%, approximately 43.4 CNY, *q* = 0.033) after FDR correction (Figure 6). Associations at lag 2 (0.104%, *q* = 0.116), lag 3 (0.075%, *q* = 0.281), and lag 6 (0.105%, *q* = 0.107) were not significant after correction. The observed decreasing trend was not statistically significant (trend estimate: −0.12% per day, *P* = 0.473).

CO: CO exposure showed a significant positive association across all lag days, though the effect magnitude was small. A log-unit increase was associated with a mean 0.036% (approximately 11.0 CNY) rise in costs, with estimates ranging from 0.032% (approximately 9.7 CNY) to 0.041% (approximately 12.4 CNY) (Figure 6). All associations remained significant after FDR correction (*q* < 0.001). Trend analysis confirmed no significant temporal variation (trend estimate: −0.00% per day, *P* = 0.983).

O_3_: No significant associations were detected between O_3_ exposure and hospitalization costs at any lag day after FDR correction (Figure 6). A log-unit increase was only associated with a non-significant mean 0.014% (approximately 4.2 CNY) rise in costs, with all 95% confidence intervals crossing zero.

### Sensitivity analysis

The robustness of our primary findings was assessed in a sensitivity analysis restricted to never-smoking patients (*n* = 1,590). The overall pattern of associations remained largely consistent, though some attenuation in effect magnitude and statistical significance was observed (Figure 7, Table 3).

SO_2_, PM_10_, and CO remained statistically significant across all lag days (*P* < 0.05). SO_2_ effects were slightly larger than in the primary analysis (range: 1.99%−3.01% per log-unit, corresponding to approximately 604.1–914.0 CNY), with the strongest effect at lag 0 (3.01%, 95% CI: 1.55%, 4.51%) and gradually declining to lag 6 (1.99%, 95% CI: 0.61%, 3.40%). PM_10_ effects ranged from 0.150% to 0.194% across lags, with peak at lag 0 (0.194%, 95% CI: 0.053%, 0.336%) and lag 3 (0.187%, 95% CI: 0.051%, 0.325%). CO showed small but consistent effects across all lags (range: 0.0345%−0.0418%).

PM_2.5_ showed significant associations only at lag 2 (0.239%, 95% CI: 0.028%, 0.452%, *P* = 0.047) and lag 3 (0.241%, 95% CI: 0.022%, 0.462%, *P* = 0.047); lag 5 was significant before correction but became non-significant after FDR adjustment (*q* = 0.066). Notably, the lag 4 and lag 6 associations observed in the primary analysis were not significant in never-smokers.

O_3_, which showed no significant associations in the primary analysis, demonstrated significant positive associations at lag 3 (0.070%, 95% CI: 0.017%, 0.123%, *P* = 0.029) and lag 5 (0.059%, 95% CI: 0.007%, 0.111%, *P* = 0.049), with lag 2 showing borderline significance (0.057%, 95% CI: 0.003%, 0.112%, *P* = 0.067), suggesting potential effect modification by smoking status.

NO_2_ was only significant at lag 0 (0.230%, 95% CI: 0.039%, 0.424%, *P* = 0.040); all other lags were non-significant, a finding consistent with the primary analysis but with reduced statistical power.

## Discussion

### Core findings and overall significance

This study fills a critical public health evidence gap by quantifying pollution-attributable AMI medical expenditure under nationally compliant low air pollution levels within China's DRG/DIP healthcare system. In this retrospective cohort study of 3,650 patients with AMI, we systematically evaluated the associations between pre-admission exposure to six major air pollutants (CO, SO_2_, PM_10_, PM_2.5_, NO_2_, and O_3_) over a lag period of 0–6 days and hospitalization costs. Our results demonstrated that short-term exposure to SO_2_, PM_2.5_, PM_10_, NO_2_, and CO was significantly associated with increased costs, whereas O_3_ showed no significant association in the primary analysis. Among these pollutants, SO_2_ exhibited the strongest effect: a log-unit increment in SO_2_ concentration corresponded to a 2.40% (approximately 729 CNY) rise in costs, followed by PM_2.5_ (0.216%, ~66 CNY), PM_10_ (0.164%, ~50 CNY), NO_2_ (0.133%, ~40 CNY), and CO (0.036%, ~11 CNY). The lag patterns were pollutant-specific: SO_2_ and CO displayed consistently significant effects across all lag days; PM_2.5_ and PM_10_ peaked at lag 4–5 days; while NO_2_ showed weaker and more fluctuating associations. In sensitivity analyses restricted to never-smokers (*n* = 1,590), the associations for SO_2_, PM_10_, and CO remained robust; however, the effects of PM_2.5_ were attenuated at several lag days, and O_3_ unexpectedly became significantly positive at certain lags, suggesting potential effect modification by smoking status. To our knowledge, this is the first study to systematically compare the lagged effects of multiple air pollutants on AMI-related hospitalization costs, specifically within the unique context of the Chinese DRG and DIP payment system, while adjusting for multiple comparisons and focusing on relatively low exposure levels, thereby filling a critical evidence gap regarding the cardiovascular economic burden attributable to ambient air pollution.

Importantly, within the current DRG/DIP payment framework—where medical expenditures are tightly aligned with disease severity and standardized clinical pathways—hospitalization costs do not fluctuate arbitrarily; rather, they primarily reflect the intensity of necessary resource utilization driven by the patient's clinical condition. Therefore, the observed cost increments attributable to air pollution are not merely financial fluctuations but constitute quantifiable evidence of pollution-induced aggravation of AMI severity, manifested as larger infarct size, increased complication rates, or greater hemodynamic instability that necessitates intensified diagnostic workup, more complex interventions, and prolonged intensive care.

### Comparison with previous literature

Our findings extend the well-established association between air pollution and adverse cardiovascular outcomes to the domain of healthcare economics. Numerous studies have confirmed that air pollution is closely associated with the incidence, mortality, and adverse cardiovascular events of AMI. In terms of incidence, short-term or long-term exposure to pollutants such as PM_2.5_, PM_10_, and NO_2_ can significantly increase the risk of myocardial infarction, including both STEMI and non-ST-segment elevation myocardial infarction (NSTEMI) (15–17). Although some findings remain inconsistent, substantial evidence supports the association between air pollution and the risk of AMI-related mortality (18, 19). Furthermore, air pollution not only affects the initial occurrence of AMI but may also elevate the risk of recurrent cardiovascular events in patients (20).

Beyond its direct impact on clinical endpoints, air pollution may also increase the medical burden on patients by exacerbating their condition and inducing complications. Existing evidence indicates that exposure to air pollution is associated with increased hospitalization costs and prolonged hospital stays among diabetic patients (6), with similar findings observed in pediatric patients with respiratory infections (7). Our study extends this line of evidence to the AMI population and, for the first time, systematically compares the effect sizes and temporal patterns of multiple pollutants. Notably, while PM_2.5_ and PM_10_ showed moderate effects with delayed peaks (lag 4–5 days), SO_2_ demonstrated both the strongest and most temporally consistent association—a pattern that has not been previously characterized in AMI cost studies.

Our findings also align with the growing body of evidence demonstrating that adverse health effects occur even at relatively low pollutant concentrations. In our study, all pollutant concentrations were consistently below the Chinese National Ambient Air Quality Standards (Grade II), yet we observed significant and economically meaningful increases in hospitalization costs. This is consistent with studies showing that short-term effects persist even when pollutant concentrations fall below the latest air quality standards established by the World Health Organization (21), reinforcing the argument that current regulatory thresholds may be insufficient to fully protect cardiovascular health.

### Biological mechanisms

The biological plausibility of our findings is grounded in the well-established pathways of oxidative stress and systemic inflammation. Air pollution exposure triggers systemic oxidative stress through excessive reactive oxygen species (ROS) generation and compromised antioxidant defenses, creating an oxidative imbalance that directly participates in myocardial infarction pathogenesis (22). Physiologically, air pollutants—including coarse, fine, and ultrafine particulate matter—rapidly impair vascular endothelial function. This endothelial dysfunction represents a critical mechanism through which pollutant signals are translated into pathological cardiovascular responses (23). The oxidative stress triggered by air pollution damages vascular endothelial cells, inducing endothelial dysfunction that subsequently promotes monocyte activation, oxidative modification of lipoproteins, and atherosclerotic plaque formation. These pathophysiological responses unequivocally accelerate atherosclerotic plaque progression, augment myocardial infarct size, and intensify coronary artery obstruction. We posit that air pollutants, through oxidative stress activation and endothelial cell impairment, may increase infarct area in acute myocardial infarction patients, consequently elevating therapeutic challenges and medical expenditures.

Among all pollutants examined, SO_2_ demonstrated the most pronounced impact on hospitalization costs, which is supported by a robust and multi-layered body of mechanistic evidence. At the molecular and cellular level, SO_2_ inhalation can induce inflammatory responses by modulating the expression of pro-inflammatory and pro-apoptotic genes in the heart and lungs. Animal studies have demonstrated that SO_2_, as an environmental pollutant, triggers a systemic inflammatory response. Moreover, short-term exposure to gaseous pollutants, including SO_2_, has been associated with an increased risk of cardiovascular disease, with the underlying mechanisms involving alterations in circulating levels of inflammatory biomarkers such as tumor necrosis factor-α (TNF-α) and interleukin-6 (IL-6) (18). Excessive SO_2_ disrupts cellular redox homeostasis, impairs the antioxidant defense system, and subsequently induces oxidative stress (24).

The unexpected finding of significant O_3_–associated cost increases observed exclusively among never-smokers in the sensitivity analysis is biologically plausible. Ozone is a highly reactive oxidant gas that exerts cardiovascular toxicity primarily through oxidative stress and systemic inflammatory pathways: upon inhalation, it reacts with components of the epithelial lining fluid in the respiratory tract to generate reactive oxygen species (ROS) and lipid oxidation products, triggering pulmonary inflammation (25) that subsequently spills over into the systemic circulation. This results in elevated levels of pro-inflammatory cytokines-including IL-6, TNF-α, and C-reactive protein (CRP) (26, 27)—and induces endothelial dysfunction, platelet hyperaggregability, and atherosclerotic plaque instability, all of which can exacerbate myocardial injury and increase the intensity of healthcare resource utilization. The apparent paradox-that O_3_ showed no significant effect in the overall population but became significant among never-smokers-can be explained by the smoking-induced tolerance hypothesis: chronic exposure to cigarette smoke induces persistent oxidative stress in the respiratory tract, which upregulates the Nrf2-mediated antioxidant defense system (including superoxide dismutase, catalase, and glutathione peroxidase), creating a preconditioning effect that renders smokers relatively less susceptible to acute ozone-induced oxidative damage (28). In contrast, never-smokers lack this adaptive upregulation and have lower baseline antioxidant capacity, making them more vulnerable to ozone-related cardiovascular consequences (29). This mechanism is consistent with previous epidemiological studies reporting stronger ozone effects in non-smokers, thereby further supporting the robustness of our findings.

Clinically, SO_2_ exposure has been directly associated with myocardial infarct size. As another significant pollutant, SO_2_ may contribute to the acute onset of ST-segment elevation myocardial infarction (STEMI) through mechanisms such as inducing systemic inflammation, altering heart rate variability, or increasing blood viscosity (30). Long-term environmental exposure to SO_2_ is also associated with an increased risk of mortality from ischemic heart disease (a 3.46% increase per 10 μg/m^3^), suggesting that SO_2_ may promote the progression of atherosclerosis and consequently affect the clinical prognosis of myocardial infarction patients by exacerbating oxidative stress and inflammatory responses (31). A study of 7,271 hospitalized patients with ischemic stroke in Chongqing, China, further demonstrated a significant positive association between short-term ambient SO_2_ exposure and hospitalization costs (32), providing direct evidence from the healthcare economic perspective that parallels our findings in AMI patients.

The distinct lag patterns observed across pollutants in our study—with SO_2_ and CO showing immediate and sustained effects, while PM_2.5_ and PM_10_ peaking at lag 4–5 days—suggest that different biological pathways and time courses may underlie their respective cardiotoxic effects. Gaseous pollutants such as SO_2_ may exert more rapid effects through direct systemic absorption and acute inflammatory responses, whereas particulate matter may act through delayed pathways involving plaque progression and secondary inflammatory cascades. This temporal heterogeneity underscores the importance of considering pollutant-specific lag structures in future exposure–response assessments.

### Public health and policy implications

The findings of this study carry significant public health and economic implications, particularly given the widespread prevalence of ambient air pollution and the substantial financial burden of cardiovascular disease. The public health importance is further underscored by China's ongoing healthcare payment reform. Under the current DRG/DIP payment system, hospitalization costs are no longer a simple aggregation of medical resource consumption but are closely tied to disease severity, clinical decision-making, and institutional cost-control efficiency (33). In this context, the significant association between air pollution exposure and increased hospitalization costs in AMI patients suggests an underlying link between air quality and disease severity, as pollution may aggravate clinical conditions, leading to higher-weighted DRG/DIP groups, prolonged stays, or increased complication rates.

Economically, our findings provide micro-level evidence on the marginal economic burden of short-term pollution exposure. A key strength of our study is its focus on a low-pollution setting—where all criteria pollutants were consistently below the Chinese National Ambient Air Quality Standards (Grade II). Despite these relatively low levels, we observed significant cost increases, particularly for SO_2_. While we do not directly estimate the cost-effectiveness of achieving stricter standards, these exposure–response coefficients offer essential input parameters for future formal health economic assessments (e.g., cost–benefit or cost-effectiveness analyses). Such assessments are necessary to determine whether the health savings from further air quality improvements would outweigh the economic costs of pollution control. This approach aligns with the Beijing experience (2013–2017), where PM_2.5_ reduction not only lowered CVD hospitalization risk but also generated an estimated annual medical cost saving of approximately 55 million yuan (34). Our results, therefore, support the rationale for conducting comprehensive economic evaluations to guide future policy calibration.

From a clinical perspective, while current AMI risk stratification systems (e.g., Global Registry of Acute Coronary Events (GRACE) / Thrombolysis in Myocardial Infarction (TIMI) scores) do not incorporate pre-admission environmental exposures, our findings suggest a potential pathway—air pollution may exacerbate infarct size or complication risk, thereby increasing resource utilization. However, translating this association into clinical practice requires additional evidence. Specifically, before integrating environmental exposure into triage or prognostic tools, its incremental clinical utility and economic value must be validated through multicenter studies and formal health economic evaluations (e.g., assessing whether incorporating exposure data improves risk prediction and resource allocation cost-effectively). Our study serves as a preliminary step by highlighting the economic signal of environmental factors, offering a testable hypothesis for future interdisciplinary research to develop standardized instruments and evaluate their added value in DRG/DIP frameworks.

### Potential impact of DRG/DIP payment reform

An important methodological consideration in this study is the potential confounding effect of China's ongoing DRG/DIP payment system reform, which was progressively implemented during our study period (2018–2024). As the reviewer correctly pointed out, changes in payment rules could affect both the accounting standards and overall levels of hospitalization costs, which raises the question of whether the observed pollution–cost associations might be confounded by secular time trends related to payment reform.

However, several factors mitigate this concern. First, DRG/DIP payment is primarily a diagnosis-based prospective payment system that sets predefined reimbursement standards for each disease group. Within the same DRG/DIP category, actual costs can still vary substantially depending on disease severity, complication rates, and procedural intensity. Our study focuses on explaining variation in costs among AMI patients within the same time period, rather than changes in average costs over time. To the extent that DRG/DIP reform affects all AMI patients uniformly within a given period, it would not confound the association between day-to-day fluctuations in air pollution and individual-level cost differences.

Second, the lag structure observed in our analysis provides further support for a causal interpretation. If the association were driven entirely by secular time trends, one would expect relatively uniform effects across lag days. Instead, we observed distinct lag patterns for different pollutants—consistent with the known temporal profiles of air pollution's cardiovascular effects—which would be difficult to explain solely by gradual policy changes.

Third, the differential effect magnitudes across pollutants are consistent with toxicological expectations. The observation that gaseous pollutants (particularly SO_2_) showed stronger associations than particulate pollutants aligns with previous literature on the health effects of air pollution in relatively low-pollution settings, and would not be expected if the results were simply an artifact of time trends.

Nevertheless, we acknowledge that we cannot fully rule out the possibility of residual confounding by secular trends, and we have added this as an important limitation in the revised manuscript. Future studies using interrupted time series designs or difference-in-differences approaches around specific policy implementation dates could help further disentangle the effects of air pollution from those of payment system reform.

### Robustness of findings

The robustness of our findings is supported by multiple analytical strategies. First, we applied the Benjamini-Hochberg procedure to control the false discovery rate for 42 comparisons, ensuring that the observed significant associations were not merely due to chance. Second, the stability of our core findings was validated through sensitivity analyses restricted to never-smokers (*n* = 1,590), in which the associations for SO_2_, PM_10_, and CO remained robust, indicating that the observed effects were not simply driven by active smoking. Third, multicollinearity was systematically assessed using Pearson correlation coefficients and VIFs. Although PM_2.5_ and PM_10_ showed a strong correlation (*r* = 0.91), all VIFs were below 10 (range: 1.30–8.42), indicating that collinearity was within acceptable limits. The moderately higher VIF values for PM_2.5_ and PM_10_ reflect their well-documented strong correlation, which is an inherent property of particulate matter; however, since we used separate single-pollutant models rather than a multi-pollutant model, collinearity between different pollutants does not bias our effect estimates. Fourth, the pollutant-specific lag patterns were consistent across the main and sensitivity analyses, lending further support to the biological plausibility of our findings. Collectively, these analytical approaches enhance the credibility of our conclusions and suggest that the observed associations are unlikely to be artifacts of multiple testing, confounding, or collinearity.

## Limitations

The methodological approach of this study has several limitations that warrant careful consideration. First, as a single-center retrospective observational study, the research population is drawn exclusively from a large chest pain center in Jiangmen, Guangdong Province. The geographical and socioeconomic characteristics of the sample are relatively limited, which may restrict the generalizability of the results to other regions. Second, exposure assessment was based on fixed-site monitoring stations, without accounting for individual-level behavioral factors such as time spent indoors, commuting patterns, or residential proximity to monitoring stations. In particular, as the reviewer noted, patients may experience reduced mobility and increased indoor time in the days preceding AMI onset, which could further contribute to exposure misclassification. This may introduce exposure misclassification, which typically biases effect estimates toward the null, suggesting that the reported associations may be underestimated. Third, although we adjusted for a comprehensive set of covariates including sex, age, smoking status, comorbidities, and meteorological factors (temperature and humidity from ERA5-Land reanalysis), several limitations remain. While VIFs indicated acceptable multicollinearity, the strong correlation between PM_2.5_ and PM_10_ (*r* = 0.91) suggests that their individual effects may not be fully separable; thus, the effect estimates for these two pollutants should be interpreted with this limitation in mind. Additionally, unmeasured confounding factors—such as socioeconomic status, medication adherence, and genetic background—may still influence the estimated associations, despite our efforts to control for measured confounders. Fourth, we did not adjust for acute clinical severity indicators of AMI, such as STEMI vs. NSTEMI classification, Killip class, number of drug-eluting stents implanted, or use of mechanical circulatory support (e.g., intra-aortic balloon pump or ECMO). These factors are important drivers of hospitalization costs in PCI-treated AMI patients, and their omission could introduce residual confounding. However, it is important to note that air pollution may increase hospitalization costs precisely by exacerbating clinical severity—for example, by triggering more extensive myocardial infarction, promoting plaque instability, or increasing the risk of complications such as cardiogenic shock. Therefore, adjusting for these intermediate variables on the causal pathway could result in over-adjustment and underestimate the true effect of air pollution on hospitalization costs. Nevertheless, we acknowledge this as an important limitation, and future studies with more detailed clinical data are needed to further disentangle these relationships. Fifth, in the sensitivity analysis restricted to never-smokers, O_3_ showed significant associations at lag 2, 3, and 5—a pattern absent in the main analysis. This discrepancy may be explained by smoking's modifying effect. Smokers chronically exposed to cigarette smoke may develop adaptive tolerance to oxidative stress, which could mask O_3_'s true effect. When smokers are removed, this masking disappears, revealing O_3_'s effect in the more susceptible never-smoker population. However, given the inconsistent lag pattern (only at lag 2, 3, and 5) and reduced statistical power in the smaller never-smoker sample (*n* = 1,590), the possibility of a false-positive finding due to multiple comparisons cannot be excluded. We therefore interpret this finding with caution and recommend confirmation in future studies. Sixth, hospitalization costs may be influenced by regional variations in pricing, reimbursement policies, and healthcare delivery patterns, which may limit comparability across different settings. Future research should adopt a multicenter prospective design, integrate individualized exposure assessment (e.g., portable monitors or spatiotemporal models), and collect more comprehensive sociobehavioral data to further validate and expand upon these findings. Seventh, our study period coincided with the progressive implementation of DRG/DIP payment reform in China, which may have influenced overall hospitalization cost levels over time. Although we believe this is unlikely to substantially bias our estimates of short-term pollution effects on individual-level cost variation, we cannot fully exclude the possibility of residual confounding by secular time trends. Future studies with more robust quasi-experimental designs are needed to confirm our findings.

## Future research directions

Future research is needed to build on these findings in several important directions. First, decomposition analyses of hospitalization cost components could help identify which specific cost categories—such as medication, device and consumables, diagnostic tests, intensive care, or procedure fees—drive the observed pollution-related cost increases, providing more granular insights into the clinical pathways involved. Second, more advanced statistical approaches, including multi-pollutant models, distributed lag non-linear models (DLNM), and Bayesian kernel machine regression (BKMR), could be applied to better characterize multipollutant interactions, non-linear exposure-response relationships, and potential threshold effects. Third, formal causal mediation analyses with detailed clinical data could help quantify the relative contributions of different pathways—such as infarct size, complication rates, or treatment intensity—through which air pollution increases medical expenditures. Finally, multicenter studies across diverse geographic and healthcare settings are needed to confirm the generalizability of our findings.

## Conclusions

In conclusion, this single-center study provides suggestive evidence that short-term pre-admission exposure to ambient air pollutants—particularly SO_2_, PM_2.5_, PM_10_, NO_2_, and CO—may be associated with increased hospitalization costs in AMI patients, even at concentrations below current national air quality standards. SO_2_ emerged as the pollutant with the strongest and most temporally consistent effect, followed by PM_2.5_ and PM_10_ with delayed peak effects. The association was robust across sensitivity analyses, and the lag patterns are consistent with distinct biological pathways. These findings extend the well-documented health effects of air pollution to the domain of healthcare economics, providing evidence that air quality improvement could have economic benefits through reduced hospital expenditures. Our results provide preliminary local evidence that may inform future air quality policy discussions and suggest a potential role for environmental factors in cardiovascular disease management that warrants further investigation. These population-based cost estimates provide preliminary local evidence for local environmental governance and cardiovascular public health resource allocation in low-pollution Chinese cities.

## Figures and Tables

**Appendix A1:** Goodness-of-fit statistics for the Gamma GLM of inpatient costs.

**Index**	Inpatient costs model
Null deviance	3,621.4
Residual deviance	2,315.6
Deviance explained (pseudo-R^2^)	(3,621.4–2,315.6)/3,621.4 = 36.1
Residual degrees of freedom (df)	473
*P-value* (*χ^2^* test)	< 0.001
Pearson *χ^2^*	2,408.3
Pearson *χ^2^/df* (overdispersion index)	2,408.3/473 = 5.09
AIC	31,278.5
BIC	31,484.3

## Data Availability

The raw data supporting the conclusions of this article will be made available by the authors upon reasonable request.
