# Supplementary material for: Short-term lagged exposure to ambient air pollutants and hospitalization costs among AMI patients: health-economic evidence from a low-pollution Chinese city with DRG/DIP standardized medical payment
Source: Front Public Health. 2026 Sep 14;14:1939605. doi: 10.3389/fpubh.2026.1939605 (PMC13617054; doi:10.3389/fpubh.2026.1939605)
Supplement: Supplementary file 1 [file Supplementary_File_1.docx]

**Supplementary Material**

*Short-term lagged exposure to ambient air pollutants and hospitalization costs among AMI patients:*

*health-economic evidence from a low-pollution Chinese city with DRG/DIP standardized medical payment*

**Table S1 | Sensitivity analysis of effect estimates for PM₂.₅ and SO₂ using varying degrees of freedom for meteorological adjustment**

| **Pollutant** | **df = 2** | **df = 3** | **df = 4** | **df = 5** | **df = 6** |
| --- | --- | --- | --- | --- | --- |
| PM₂.₅ | 0.201% | 0.192% | 0.186% | 0.186% | 0.188% |
| SO₂ | 1.76% | 1.76% | 1.72% | 1.71% | 1.72% |

Effect estimates are presented as the percentage change in hospitalization cost per one log-unit increase in pollutant concentration. Models were fitted using a Gamma GLM with log link, adjusting for sex, age, six comorbidities (hypertension, diabetes mellitus, COPD, pneumonia, dyslipidemia, and chronic kidney disease), season, day of week, smoking status, and meteorological factors (temperature and relative humidity) specified as natural cubic splines with degrees of freedom (df) ranging from 2 to 6. All effect estimates remained highly stable across different df specifications, with maximum fluctuations of less than 8%, confirming the robustness of the primary findings.

**Table S2 | Sensitivity analysis: effect estimates before and after adjustment for length of hospital stay (LOS)**

| **Pollutant** | **Lag** | **Without LOS % change** | **Without LOS 95% CI** | **Without LOS P-value** | **With LOS % change** | **With LOS 95% CI** | **With LOS P-value** | **Attenuation (%)** |
| --- | --- | --- | --- | --- | --- | --- | --- | --- |
| SO₂ | 0 | 2.59% | (1.77, 3.42) | <0.001 | 2.47% | (1.78, 3.17) | <0.001 | 4.5% |
| SO₂ | 1 | 1.88% | (1.24, 2.52) | <0.001 | 1.45% | (0.92, 2.00) | <0.001 | 22.5% |
| SO₂ | 2 | 1.69% | (1.13, 2.24) | <0.001 | 1.08% | (0.62, 1.55) | <0.001 | 35.8% |
| SO₂ | 3 | 1.60% | (1.05, 2.15) | <0.001 | 1.11% | (0.65, 1.58) | <0.001 | 30.4% |
| SO₂ | 4 | 1.52% | (0.98, 2.06) | <0.001 | 1.14% | (0.68, 1.60) | <0.001 | 25.0% |
| SO₂ | 5 | 1.41% | (0.93, 1.90) | <0.001 | 1.12% | (0.71, 1.53) | <0.001 | 21.0% |
| SO₂ | 6 | 1.62% | (1.08, 2.15) | <0.001 | 1.33% | (0.88, 1.78) | <0.001 | 17.8% |
| PM₂.₅ | 0 | 0.19% | (0.04, 0.34) | 0.012 | 0.17% | (0.05, 0.30) | 0.006 | 7.7% |
| PM₂.₅ | 1 | 0.19% | (0.04, 0.33) | 0.012 | 0.15% | (0.03, 0.27) | 0.016 | 19.3% |
| PM₂.₅ | 2 | 0.16% | (0.02, 0.30) | 0.021 | 0.16% | (0.04, 0.28) | 0.008 | 2.0% |
| PM₂.₅ | 3 | 0.16% | (0.02, 0.30) | 0.024 | 0.15% | (0.03, 0.27) | 0.013 | 7.3% |
| PM₂.₅ | 4 | 0.25% | (0.11, 0.39) | <0.001 | 0.19% | (0.07, 0.31) | 0.001 | 23.4% |
| PM₂.₅ | 5 | 0.24% | (0.10, 0.38) | 0.001 | 0.19% | (0.07, 0.31) | 0.001 | 17.7% |
| PM₂.₅ | 6 | 0.16% | (0.03, 0.29) | 0.019 | 0.17% | (0.06, 0.28) | 0.003 | -5.7% |

Effect estimates are presented as the percentage change in hospitalization cost per one log-unit increase in pollutant concentration. Models were fitted using a Gamma GLM with log link, adjusting for sex, age, six comorbidities (hypertension, diabetes mellitus, COPD, pneumonia, dyslipidemia, and chronic kidney disease), season, day of week, smoking status, and meteorological factors (temperature and relative humidity, natural cubic splines with df = 3). "Without LOS adjustment" refers to the primary analysis model; "With LOS adjustment" refers to the model with length of hospital stay added as an additional covariate. Attenuation (%) was calculated as (1 − effect_with_LOS / effect_without_LOS) × 100. All statistically significant associations in the primary analysis remained significant after adjustment for LOS, indicating that the majority of pollution-associated cost increases reflect higher per-day resource utilization rather than simply prolonged hospital stay.

**Table S3 | Variance Inflation Factor (VIF) values for six criteria air pollutants**

| **Pollutant** | **VIF** |
| --- | --- |
| PM₂.₅ | 7.89 |
| PM₁₀ | 8.42 |
| SO₂ | 1.30 |
| NO₂ | 3.05 |
| CO | 2.14 |
| O₃ | 1.46 |

VIF values were calculated based on natural log-transformed daily mean concentrations of six air pollutants (PM₂.₅, PM₁₀, SO₂, NO₂, CO, and O₃) during 2018–2024. A VIF value < 10 is generally considered indicative of no severe multicollinearity. The moderately higher VIF values for PM₂.₅ and PM₁₀ reflect their strong correlation (r ≈ 0.9), which is an inherent property of particulate matter. Since separate single-pollutant models were used in the primary analysis, collinearity between different pollutants does not bias the effect estimates.

**Table S4 | Sensitivity analysis: associations between air pollutant exposure and hospitalization costs after excluding the COVID-19 pandemic period (2020–2022)**

| **Pollutant** | **Lag** | **% Change** | **95% CI** | **P-value** |
| --- | --- | --- | --- | --- |
| SO₂ | 0 | 3.16 | (2.25, 4.08) | <0.001 |
| SO₂ | 1 | 2.26 | (1.57, 2.96) | <0.001 |
| SO₂ | 2 | 2.05 | (1.45, 2.65) | <0.001 |
| SO₂ | 3 | 1.82 | (1.22, 2.42) | <0.001 |
| SO₂ | 4 | 1.73 | (1.15, 2.32) | <0.001 |
| SO₂ | 5 | 1.58 | (1.06, 2.10) | <0.001 |
| SO₂ | 6 | 2.17 | (1.60, 2.75) | <0.001 |
| PM₂.₅ | 0 | 0.368 | (0.190, 0.548) | <0.001 |
| PM₂.₅ | 1 | 0.379 | (0.202, 0.556) | <0.001 |
| PM₂.₅ | 2 | 0.333 | (0.157, 0.510) | <0.001 |
| PM₂.₅ | 3 | 0.337 | (0.159, 0.515) | <0.001 |
| PM₂.₅ | 4 | 0.440 | (0.265, 0.615) | <0.001 |
| PM₂.₅ | 5 | 0.491 | (0.312, 0.671) | <0.001 |
| PM₂.₅ | 6 | 0.372 | (0.201, 0.543) | <0.001 |
| PM₁₀ | 0 | 0.275 | (0.163, 0.387) | <0.001 |
| PM₁₀ | 1 | 0.294 | (0.184, 0.404) | <0.001 |
| PM₁₀ | 2 | 0.261 | (0.150, 0.372) | <0.001 |
| PM₁₀ | 3 | 0.281 | (0.165, 0.396) | <0.001 |
| PM₁₀ | 4 | 0.356 | (0.245, 0.468) | <0.001 |
| PM₁₀ | 5 | 0.348 | (0.237, 0.459) | <0.001 |
| PM₁₀ | 6 | 0.275 | (0.168, 0.383) | <0.001 |
| NO₂ | 0 | 0.262 | (0.098, 0.427) | 0.002 |
| NO₂ | 1 | 0.255 | (0.092, 0.418) | 0.002 |
| NO₂ | 2 | 0.201 | (0.039, 0.364) | 0.015 |
| NO₂ | 3 | 0.189 | (0.025, 0.352) | 0.024 |
| NO₂ | 4 | 0.313 | (0.151, 0.475) | <0.001 |
| NO₂ | 5 | 0.313 | (0.151, 0.476) | <0.001 |
| NO₂ | 6 | 0.245 | (0.089, 0.402) | 0.002 |
| CO | 0 | 0.050 | (0.038, 0.062) | <0.001 |
| CO | 1 | 0.045 | (0.033, 0.057) | <0.001 |
| CO | 2 | 0.040 | (0.028, 0.053) | <0.001 |
| CO | 3 | 0.041 | (0.028, 0.053) | <0.001 |
| CO | 4 | 0.049 | (0.037, 0.061) | <0.001 |
| CO | 5 | 0.049 | (0.037, 0.061) | <0.001 |
| CO | 6 | 0.047 | (0.034, 0.059) | <0.001 |
| O₃ | 0 | 0.034 | (-0.016, 0.085) | 0.182 |
| O₃ | 1 | 0.014 | (-0.034, 0.063) | 0.560 |
| O₃ | 2 | 0.035 | (-0.011, 0.081) | 0.135 |
| O₃ | 3 | 0.041 | (-0.004, 0.086) | 0.075 |
| O₃ | 4 | 0.062 | (0.016, 0.107) | 0.008 |
| O₃ | 5 | 0.068 | (0.024, 0.112) | 0.002 |
| O₃ | 6 | 0.051 | (0.007, 0.095) | 0.024 |

Values represent the percentage change in hospitalization costs per one natural log-unit increase in pollutant concentration, estimated using generalized linear models with Gamma distribution and log link. Models were adjusted for sex, age, comorbidities (diabetes mellitus, hypertension, COPD, pneumonia, hyperlipidemia, and renal insufficiency), season, day of week, smoking status, and same-day temperature and relative humidity (natural cubic splines, df = 3). This sensitivity analysis excludes data from the COVID-19 pandemic period (2020–2022) and includes only data from 2018–2019 and 2023–2024. P-values are raw (unadjusted).
